# Effect of Biodegradable Binder Properties and Operating Conditions on Growth of Urea Particles in a Fluidized Bed Granulator

**DOI:** 10.3390/ma12142320

**Published:** 2019-07-20

**Authors:** Ehsan Zhalehrajabi, Kok Keong Lau, Ku Zilati Ku Shaari, Seyed Mojib Zahraee, Seyed Hadi Seyedin, Babar Azeem, Azizah Shaaban

**Affiliations:** 1 Department of Chemical Engineering, Universiti Teknologi PETRONAS, Bandar Seri Iskandar 32610, Perak, Malaysia; 2 CO2 Research Centre (CO2RES), Institute of Contaminant Management, Universiti Teknologi PETRONAS, Bandar Seri Iskandar 32610, Perak, Malaysia; 3 School of Engineering, Department of Mechanical, Manufacturing, and Mechatronics, RMIT University, Melbourne 3000, Australia; 4 Faculty of Manufacturing Engineering, Universiti Teknikal Malaysia Melaka, Hang Tuah Jaya, 76100 Melaka, Malaysia

**Keywords:** agglomeration, fluidized bed, granulation, particle growth, starch binder, urea

## Abstract

Granulation is an important step during the production of urea granules. Most of the commercial binders used for granulation are toxic and non-biodegradable. In this study, a fully biodegradable and cost-effective starch-based binder is used for urea granulation in a fluidized bed granulator. The effect of binder properties such as viscosity, surface tension, contact angle, penetration time, and liquid bridge bonding force on granulation performance is studied. In addition, the effect of fluidized bed process parameters such as fluidizing air inlet velocity, air temperature, weight of primary urea particles, binder spray rate, and binder concentration is also evaluated using response surface methodology. Based on the results, binder with higher concentration demonstrates higher viscosity and higher penetration time that potentially enhance the granulation performance. The viscous Stokes number for binder with higher concentration is lower than critical Stokes number that increases coalescence rate. Higher viscosity and lower restitution coefficient of urea particles result in elastic losses and subsequent successful coalescence. Statistical analysis indicate that air velocity, air temperature, and weight of primary urea particles have major effects on granulation performance. Higher air velocity increases probability of collision, whereby lower temperature prevents binder to be dried up prior to collision. Findings of this study can be useful for process scale-up and industrial application.

## 1. Introduction

Fluidized bed granulation is used in a range of industries such as pharmaceuticals, foods, detergents, and fertilizer products. In a fluidization process, granular material is subjected as a solid state to behave like a fluid. In agrochemical industry, the synthesized fertilizer is usually converted into particulate material either through granulation or prilling. Since granules possess better characteristics than prills, granulation becomes the preferred route for urea fertilizer production [1]. Granulation is an enlargement process that combines fine particles into a larger mass of aggregates by spraying a binder solution onto the dry powder bed [2]. Typically, a powder bed is agitated by air and granules are formed with the addition of a liquid binder. Despite the widespread use of fluidized bed granulation, prediction of granule size distribution is still a huge challenge. The principal rate processes influencing the granule size distribution during granulation can be summarised as wetting and nucleation, consolidation and growth, and attrition and breakage [3]. The knowledge of rates of these processes can allow the prediction of granule size distribution. In general, most of literature reports cover experimental studies pertaining to the role of material properties and process conditions on the characteristics of product granules. 

There are three types of commercial binders; natural, synthetic, and semi synthetic. Recent studies of Dürig and Karan [4] cover a vast range of binders that are used in wet granulation. Previous research focused mostly on the use of synthetic binders such as polyethylene glycol (PEG). Particle size distribution (PSD) was studied using one-dimensional (1D) population balance model (PBM) for the granulation of CaCO_3_ and lactose using PEG and hydroxypropyle as the binder solution [5]. Box-Behnken method was used to analyse the effect of atomizing air flow rate, binder spray rate and binder volume on granulation performance of lactose using PEG 4000 mix with indomethacin in a fluidized bed granulator [6]. Effect of spray rate, droplet size, and operating temperature was investigated on PSD of substrate particles in a fluidized bed granulator using PEG 1500 as a binder. The PSD was modelled using 1D PBM and the hydrodynamics of fluidized bed was predicted using Computational Fluid Dynamics (CFD) modelling [7,8]. The aggregation efficiency of CaCO_3_ particles in a high-shear granulator was estimated using PBM and particle image velocimetry. PEG 4000 was used as a binder in this case [9]. The prediction of PSD by solving PBM equations using different aggregation kernel and breakage models was reported for particles sprayed by PEG 1500 in a high-shear granulator [10,11]. Another study depicts the effect of binder mass, porosity, and primary particle size on PSD of CaCO_3_ particles in a high-shear mixer using PEG as a binder [12]. PEG has a low melting point. In a warm environmental condition, the cold storage must be provided to avoid melting or sticking of PEG particles with each other. Furthermore, there are environmental concerns for the use of PEG. The use of polymeric binder is also reported in literature. Ennis et al. [13] utilized polymeric binders such as sodium carboxymethyl cellulose (CMC), polyvinylpyrolidone, and hydroxyethyl cellulose (Klucel) to investigate viscous Stokes number. They classified coalescence mechanism based on collisional dissipation of relative kinetic energy. Heinrich et al. [14] also used CMC as a binder in a continuous spray fluidized bed to determine PSD by considering air flow rate and attrition on particles. Another study [15] reported the effect of operating parameters and binder properties on PSD using different concentrations of CMC in a coating and granulation process. Tardos et al. [16] investigated the effect of binder properties on granulation process using CMC and Carbowax as binders. Despite an acceptable performance by synthetic binders, use of natural binders has recently got popular to diminish the adverse effects of synthetic materials on environment and reduce the overall cost of production. 

The formation of liquid bridge between two particles using different types of binders is reported in the literature. Studies of Kivikero [17], Patel et al. [18] and Reynolds et al. [19] show the effect of binder physical properties on granule size and strength. Since the binder properties such as viscosity, surface tension, droplet contact angle, and droplet penetration time significantly influence the granulation performance, their effect on granulation must be thoroughly and systematically investigated. Due to extensive mixing of particles and excellent heat and mass transfer features, a fluidized bed granulator is a promising choice to study the granulation process. In addition to binder properties, the fluidized bed operating conditions such as fluidizing air velocity, air temperature, and spray rate etc. play a critical role in the growth mechanism of particles during granulation. In multiphase flow and fluidization studies, flow regime and air inlet temperature have a significant effect on particles size distribution. Morl et al. [20], Nishii and Horio [21], and Saleh and Guigon [22] investigated growth mechanism of particles in fluidized bed reactor at various operating conditions. In fluidized bed granulator, one of the important components is the spray nozzle. The role of a spray nozzle is to shower the binder droplets onto powder mass in as uniform a layer as possible. Other than nozzle type and atomizing air pressure, the droplet size of binder solution hugely depends on liquid viscosity and surface tension [23]. Ennis et al. [24] and Ennis and Robert [25] investigated effect of binder viscosity on pendular liquid bridge. They determined that strength of bridge related to magnitude of viscous force and capillary force as well. Location of nozzle, spray rate, and atomizing pressure have been investigated and optimum conditions based on mass of primary particles (substrate) and size of chamber to achieve a good granulation performance are reported in literature [26]. 

Response surface methodology (RSM) is used to optimize process variables to achieve targeted responses. RSM provides adequate and reliable response metrics by developing a mathematical model with a ‘best fit’ for the obtained data. The results are fitted to the model for each response and the model significance is evaluated by the analysis of variance (ANOVA) [27]. So far, a very few studies have reported RSM based experiments to investigate the interactive effect of binder properties and fluidized bed operating parameters on the performance of urea granulation using a fully biodegradable binder in a pulsed spray fluidized bed granulator.

In this study, a new biodegradable (natural) binder is developed using starch and urea. Due to limited work on biodegradable binders, the effect of binder physical properties such as viscosity, surface tension, contact angle, droplet penetration time, and liquid bridge bonding force is investigated on weight gain of granulated particles (particle growth). Fluidized bed granulation machine with top spray orientation is used for the granulation of substrate urea powder (primary particles). The interactive effect of air inlet velocity, air inlet temperature, spray rate, primary loaded particle, and binder viscosity is investigated on granulation performance using RSM. 

## 2. Materials and Methods

### 2.1. Materials

Urea particles, supplied by Hap Seng SDN BHD., Kuala Lumpur, Malaysia, are used as substrate material to study the growth rate of granulated particles. The average size of urea granules provided by supplier is 3.5 mm. These granules were milled in a grinder and subsequent urea powder was subjected to sieve analysis such that the size of primary urea particles ranged from 50 to 500 µm. Since density of urea is 1.3 g/cm^3^, the hydrodynamic behavior of these urea particles in fluidized bed lies in between Group A and Group B of the Geldart classification. [28]. A smooth fluidization of the substrate particles is predicted when they are in between Group A and Group B of the Geldart classification [28]. These substrate particles were weighed and charged into a fluidized bed granulator (FBG). Wheat starch provided by Synerchem Food Processing Ind. SDN. BHD. (Shah Alam, Malaysia) was used to prepare the binder solution by the addition of calculated amounts of urea and starch into the pre-weighed amount of water under constant stirring and heating up to 70 °C to achieve complete dissolution of the materials. 

The binder solution prepared with a combination of urea, starch, and water (USW) is completely biodegradable. Different concentrations of the binder solution are illustrated in Table 1. A concentration of USW binder higher than 3 *w*/*w*% causes crystallization and less than 1 *w*/*w*% results in weaker cohesive force. 

### 2.2. Methods

#### 2.2.1. Binder Properties 

One of the important factors in granulation process is the bonding between substrate particles. When two wet particles collide with each other, a liquid bridge is formed between them. The wetting of substrate particles by the impingement of binder droplet and subsequent liquid bridge formation and growth of particles to form granulates depend on several properties of the binder solution such as viscosity, density, surface tension, contact angle, penetration time, and liquid bridge bonding forces. Methods to evaluate these binder properties are detailed as follows. 

##### Binder Viscosity and Density

Viscous forces dominate in particle-particle interactions [19]. Viscosity has direct effect on penetration time and dynamic (viscous) force. Several experimental and theoretical works show that the extent of agglomeration increases with increasing liquid viscosity [24,29,30,31]. In current work, binder density was measured by Anton Paar DMA 4500M (Anton Paar GmbH, Graz, Austria) and viscosity was measured by rotational viscometer, Anton Paar MCR 302 (Anton Paar, Graz, Austria). 

##### Binder Surface Tension

Surface tension affects the strength of capillary (static) forces [19] and liquid bridge between two particles [32]. Due to strength of the capillary bond in granulation, a minimum surface tension is required to granulate particles of a certain size [33]. We determined surface tension of the binder by pendant-drop method using DataPhysics Optical Contact Angle (OCA) 15EC device (DataPhysics Instruments GmbH, Filderstadt, Germany). In this method, a needle (Nordson SNS-D 051/025, Westlake, OH, USA) with inner diameter of 0.525 mm was used to produce a droplet. 

##### Binder Contact Angle

Droplet contact angle affects wetting behavior of binder liquid on the surface of particles [19]. The quality and morphology of the final granulated product depends on wetting parameters of the binder. Wettability describes the ability of a liquid to spread over the surface of a solid material. In this study, droplet contact angle was measured by Sessile Drop technique using DataPhysics OCA 15EC device. Urea powder was pelletized and used as a solid surface. Needle (Nordson SNS-D 051/025) with inner diameter of 0.525 mm was used to produce the droplet. 

##### Droplet Penetration Time

Penetration time refers to the time taken by binder droplet to penetrate into the surface of substrate particle. Penetration time affects the coalescence yield and granulation performance. An extended penetration time facilitates the successful impingement of other substrate particles by the development of liquid bridges thereby giving rise to the particles’ growth [34]. In this study, a carefully metered single drop is placed on a cautiously prepared powder surface to determine time for the complete penetration of drop. High speed camera (Phantom Miro M320S, Vision Research ® Inc, Wayne, NJ, USA) was used to record droplet penetration time.

##### Liquid Bridge Bonding Forces

**Figure 1:**
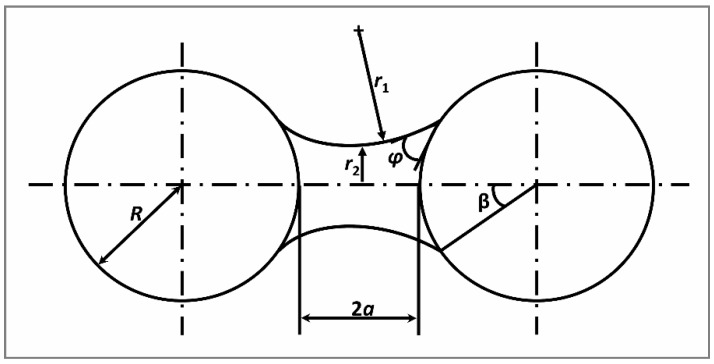
Binary agglomeration due to liquid bridge.

Liquid bridge bonding forces affect the strength of agglomerated particles. Strong bonding force prevents the breakage of agglomerate particles and increases the growth rate. The solid bridge is formed when water evaporates from the binder solution. A liquid bridge is constituted by the dynamic and static forces such that both of these forces are energy dissipative [35]. In this study, we calculated the liquid bridge bonding force by the addition of static and dynamic forces. The static liquid bridge force, also called capillary force, is the sum of surface tension force and suction pressure force as described by Equation (1) [36,37].

(1) $$F_{\mathrm{stat}}=2\pi r_{2}\gamma +\pi r_{2}^{2}\Delta P$$

where ∆*P* is given by the Young-Laplace equation (Equation (2)).

(2) $$\Delta P=\gamma \left(\frac{1}{r_{1}}-\frac{1}{r_{2}}\right)$$

where γ is surface tension of the binder liquid. Static (capillary) force for two identical touching spherical particles is illustrated in Figure 1 [38,39].

In Figure 1, *R*, *r_1_*, and *r_2_* refer to single particle radius, circle radius, and minimum distances between bridge edges, respectively. We used another relationship to calculate the static force by Rumpf’s equation (Equation (3)), compared the resulting values with the results obtained from Equation (1), and calculated the percentage error. In both Equations (1) and (3), the bond strength due to a static liquid bridge can be related to the liquid surface tension, γ, and solid-liquid contact angle, *φ,* as portrayed in Equation (3).

(3) $$F_{\mathrm{stat}}=4\pi \gamma R^{2}\sin^{2}\beta +2\pi \gamma R\sin\left(\beta +\varphi \right)$$

where *β* is the liquid filling angle that depends on volume of the liquid bridge [22]. 

The dynamic force in the liquid bridge is relative to the movement of two particles. When two particles collide, either they join together or rebound. Equation (4) represents the dynamic force between two particles [35,36].

(4) $$F_{\mathrm{dyn}}=\frac{6\pi \mu R^{2}U}{a}$$

where *µ*, *U*, and *a* are liquid binder viscosity, average velocity of particles, and half distance between coalescing particles, respectively.

To estimate sensitivity of pendular bridge to viscous or capillary forces or both, Enni’s finding emphasized that capillary viscous number (*Ca*_vis_) that is a measure of relative magnitude of viscous forces to capillary forces, can be used to estimate the strength of a dynamic pendular bridge [13]. In this study, we estimated *Ca*_vis_ by dividing Equation (4) with Equation (1). Scanning electronic microscope (SEM), TM3030-HITACHI (Tokyo, Japan), is used to determine particles size and bridge length. Since it was extremely difficult to identify the exact bonding point on the surface of urea particles, we used 2 mm spherical glass beads (Sigma-Aldrich, St. Louis, MI, USA) to determine capillary and viscous forces. 

**Figure 2:**
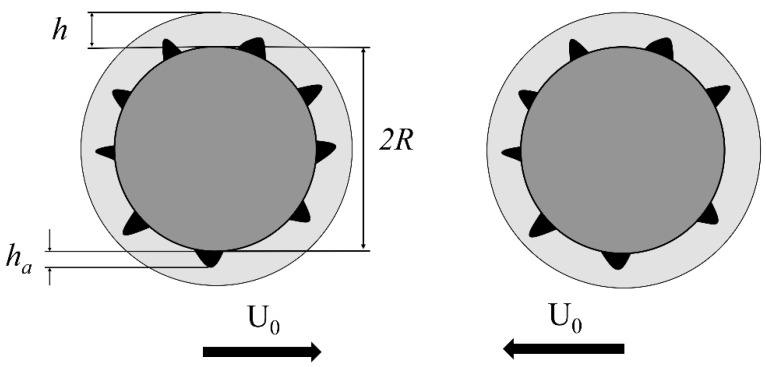
Schematic of two colliding particles covered by liquid binder layer of thickness *h* and the asperities length of *h_a_*.

The first stage of binary agglomeration is coalescence. Two colliding particles coalesce if the liquid bridge formed is strong enough to prevent rebound after collision. The particle pair may be broken by colliding to other particles and successful coalescence consider as an unsuccessful agglomeration. In order to establish regimes of granulation, Ennis et al. defined the viscous Stokes number, Stv, as the ratio of the relative kinetic energy between colliding particles to the viscous dissipation brought about by pendular bonds [22]:

(5) $${St}_{v}=\frac{8\rho RU_{0}}{9\mu }$$

where U_0_ is the relative velocity of particles, *ρ*_p_, the particle density and *µ* the viscosity of the binding liquid. To calculate *St*_v_ the value of interparticle velocity, U_0_, should be measured and this reflects the effect of break-up forces imposed by granulation system. A critical viscous Stokes number *St*_v_* must be surpassed for rebound of colliding particles to occur:

(6) $${St}_{v}^{*}=\left(1+\frac{1}{e}\right)\ln\left(\frac{h}{h_{a}}\right)$$

where *e* is the particle coefficient of restitution, *h* the thickness of the binder layer and *h_a_* a measure of the particle’s surface asperities (see Figure 2). Coefficient of restitution was measured for urea particles and its value is 0.188. 

Three granulation regimes were defined in terms of the magnitude of *St*_v_ in comparison with $St_{v}^{*}$:

${St}_{v}\leq {St}_{v}^{*}$ non inertial regime (all collisions successful)
${St}_{v}\approx {St}_{v}^{*}$ inertial regime (some collisions successful)
${St}_{v}\geq {St}_{v}^{*}$ coating regime (no collisions successful)

High value of $St_{v}^{*}$ means that the two colliding particles may be larger or their velocities may be higher, and still successfully coalesce [22,35].

#### 2.2.2. Fluidization Experiments for Urea Granulation

##### Primary Particle Size

Primary particles (substrate) size plays an important role to determine the amount of binder required for granulation. Relatively more quantity of liquid is used for a lower primary particle size due to extended surface area. To the contrary, less quantity of liquid is required to obtain an identical average granule size when a larger particle size is used for granulation [40]. However, the liquid requirement is also influenced by other factors such as porosity of substrate particles. The primary particle size also influences the critical viscosity that is required to promote granule growth [41]. To prevent complete breakage, a higher binder viscosity is necessary when the primary particle size of the feed material is larger [19]. In this study, we determined the primary particle size by sieve analysis. A set of sieves (20, 50, 100, 212, 500, and 1000 µm) supplied by Retsch GMBH, was used to evaluate particle size distribution.

##### Top-Spray Fluidized Bed Granulation

**Figure 3:**
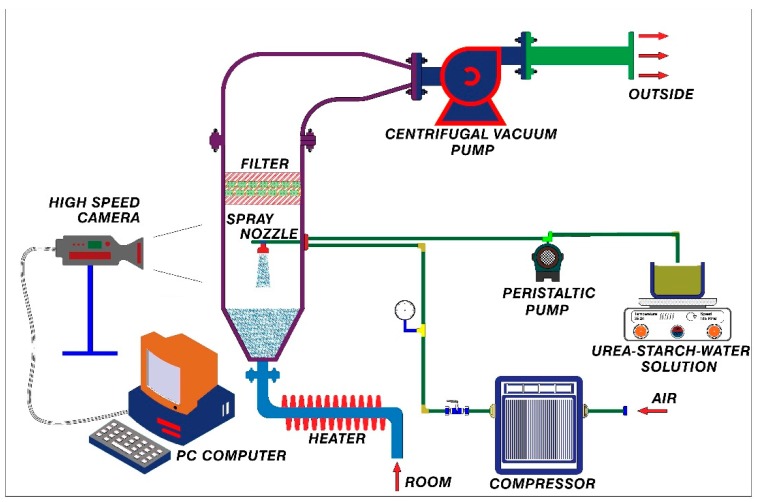
Schematic diagram of fluidized bed granulator.

Granulation experiments were carried out in a lab-scale batch fluidized bed granulator (Changzhou Jiafa Granulation Drying Equipment Co., Ltd, Changzhou, China). The schematic diagram for the experimental setup is shown in Figure 3. The product chamber is a conical cylinder made from stainless steel and plexi glass. The height of the container is 55 cm with inner diameters of 10 cm and 24.5 cm at the bottom and top respectively. The air distributor is a 35-mesh stainless steel plate at the bottom of the product container. The inlet air is preheated by an electrical heater before entering into the bed for each experiment. Air inlet velocity is adjusted by changing the rotational speed of the blower through an inverter device that changes the inlet electrical frequency to the blower’s driver. In this study, air inlet velocity of 35 cm/s is considered as an average velocity for fluidization of particles with *d_p_* = 250 µm. But experimentally, particles size distribution is between 50–500 µm. The binder spray nozzle is a movable, external mixing two-fluid spray nozzle placed 28 cm above the distributor plate. The spray from nozzle is counter current to the upward fluidizing air flow. A peristaltic pump (Longer pump BT100-2J, Longer Precision Pump Co., Ltd., Hebei, China) transports the binder solution from a reservoir into the spray nozzle. The volumetric flow rate of the binder solution is adjusted via setting the rotational speed of the peristaltic pump.

##### Test Procedure

A pre-weighed amount of urea powder was charged into the fluidized bed chamber and the stream of hot air with predetermined air velocity and temperature was set to achieve the powder fluidization. The fluidizing air velocity was determined by a pitot tube. An air compressor atomized the binder solution at a pressure of 1 barg in all the experiments. The spraying of binder solution was done in pulse mode with 2 s of spray followed by a 5 s cut-off situation. The cut off situation was achieved by closing the atomizing air through an automatic valve actuator and switching off the peristaltic pump simultaneous. The granulation test was continued until the agglomerated granules became too large to fluidize any further. The time required to achieve this stage ranged between 6–15 min. depending upon the operating conditions.

##### Sampling and Granule Size Measurement

The granule size distribution of the final product was determined by sieve analysis. A set of sieves (500, 1000, 2000, 4000, and 5000 µm) in combination with the Retsch AS 200 Sieve Shaker (RETSCH, Haan, Germany) was used for size distribution analysis. Before the particle size analysis, the final granules were dried in an oven at 60 °C for 24 h. The dried granules were transferred into the pre-weighed sieves and allowed to shake at an amplitude of 1 mm for 10 min. The sieves were re-weighed to determine the weight of granules with particle size ranging between 2000–4000 µm (2–4 mm).

##### Design of Experiments

Design of experiments (DOE) is a systematic method to determine the relationship between factors affecting a process and to find the cause-and-effect relationship [42,43]. Design of Expert^®^ version 10 has been used for design of experiment and data analysis. In this work, Full Factorial design is selected to evaluate the effect of fluidized bed operating parameters on the growth of granulated particles. Table 2 shows the factors and their levels where each factor has a high (+), center (0) and low (−) level. The low level and high level magnitudes of the operating parameters (factors) were chosen based on the preliminary trial runs.

## 3. Results and Discussion

### 3.1. Binder Properties

#### 3.1.1. Binder Density and Viscosity 

The densities and viscosities of the USW binder with different concentrations is presented in Table 3. Adding more urea and starch into water changes the composition of particles in a given volume of solution. This results in change of mass per unit volume of the solution (density). By increasing the amount of starch, solution density decreases due to the development of strong crosslink network through crosslinking reactions with –OH, –NH_2_, and –SH groups [44]. Since urea and water have –NH_2_ and –OH groups respectively, they can crosslink with starch. Besides, starch also penetrates into the intermolecular gaps of water molecules and fills this gap by molecules’ chains of starch and urea such that volume of the solution increases per unit mass.

**Figure 4:**
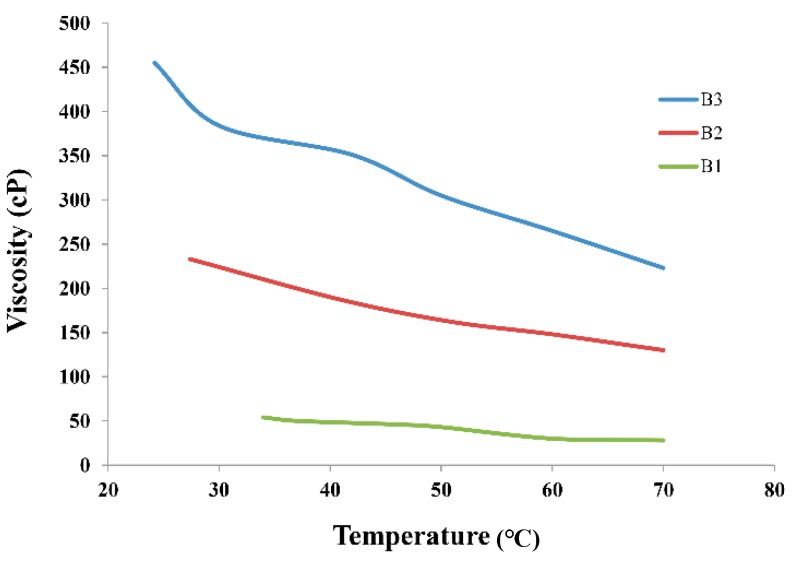
Viscosity vs. temperature for different binder concentrations (B1, B2, and B3).

By increasing starch content, viscosity increases due to the formation of higher number of crosslink chains between urea-starch-water such that the solution tends to gelatinize [44]. A plot of viscosity vs. temperature (Figure 4) indicates that binder viscosity decreases with increasing temperature. 

During fluidization, viscosity of the binder droplets changes when they come across hot air in the fluidized bed chamber. Due to a temperature gradient at different locations inside the fluidized bed chamber, it is difficult to measure viscosity of the droplets at impact point between droplet and the particle surface. In this study, we found that increase in binder viscosity leads to a maximum extent of granulation in the nucleation and compaction regime [45]. Faster growth was observed in the coalescence stage. Increase in binder viscosity led to the increase in average granule size and penetration time. However, higher viscosity and adhesion force between binder and particles made particles bigger and probability of attrition breakage is minimized. The partial dissolution of substrate particles into binder droplets and increase in temperature over the course of experiment change the droplet viscosity. In a drum granulator, increase in binder viscosity decreases the rate of intra-granular porosity [33].

For many granulating systems, however, pre-wetting cannot be used either because the binder reacts with the powder (as in case of detergent manufacture) or the binder solidifies if temperature decreases (e.g., in the production of pharmaceutical products where high molecular weight PEGs are used). This allows dissociation of nucleation from growth phenomena. Since nucleation affects the initial distribution of binder within the system, the pre-wetted powder would not be representative of an industrial process. Depending on the primary particle size, a certain viscosity must be exceeded in order to obtain granule growth [41,46]. When large primary particles were granulated with a low-viscosity binder, granule growth was limited [19].

#### 3.1.2. Binder Surface Tension

Table 4 represents surface tension of the USW binder with different concentrations. Results for binder surface tension indicate that B3 has a lower surface tension. A decrease in binder surface tension decreases the dynamic yield-stress of granules [19,33]. 

However, when surface tension varies for more viscous binder, the binder viscosity dominates the yield-stress behavior [19,47]. Decrease in surface tension led to increase in the minimum intra-granular porosity [33].

#### 3.1.3. Binder Contact Angle

**Figure 5:**
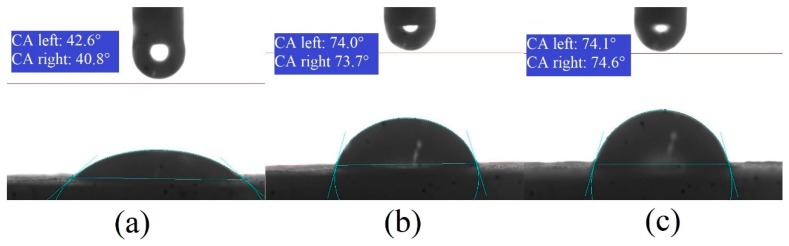
Contact angle for USW binder with different concentrations: (**a**) B1, (**b**) B2 and (**c**) B3.

Figure 5 represents contact angles for USW binder solutions with different concentrations. B1 and B3 have the lowest and highest contact angles respectively. It is observed that contact angle increases by increasing viscosity. Due to the increase in weight percentage of urea and starch in B3, the intra-molecular cohesive forces between liquid molecules dominate the liquid-solid adhesive force such that liquid droplets observe a delayed spreading. This results in a larger droplet lamella and subsequently higher contact angle that increases the penetration time. A longer penetration time refers to an extended retention period of droplet on the substrate surface that enhances the possibility of successful impingement and coalescence between the particles. 

Lower contact angle implies that the droplet wetting, spreading, and penetration into the particle surface takes place in a relatively shorter period of time. To the contrary, a larger contact angle implies that liquid does not wet the surface quickly and tends to form beads [22]. Table 5 represents the contact angles for different concentrations of the USW binder. 

#### 3.1.4. Droplet Penetration Time

**Figure 6:**
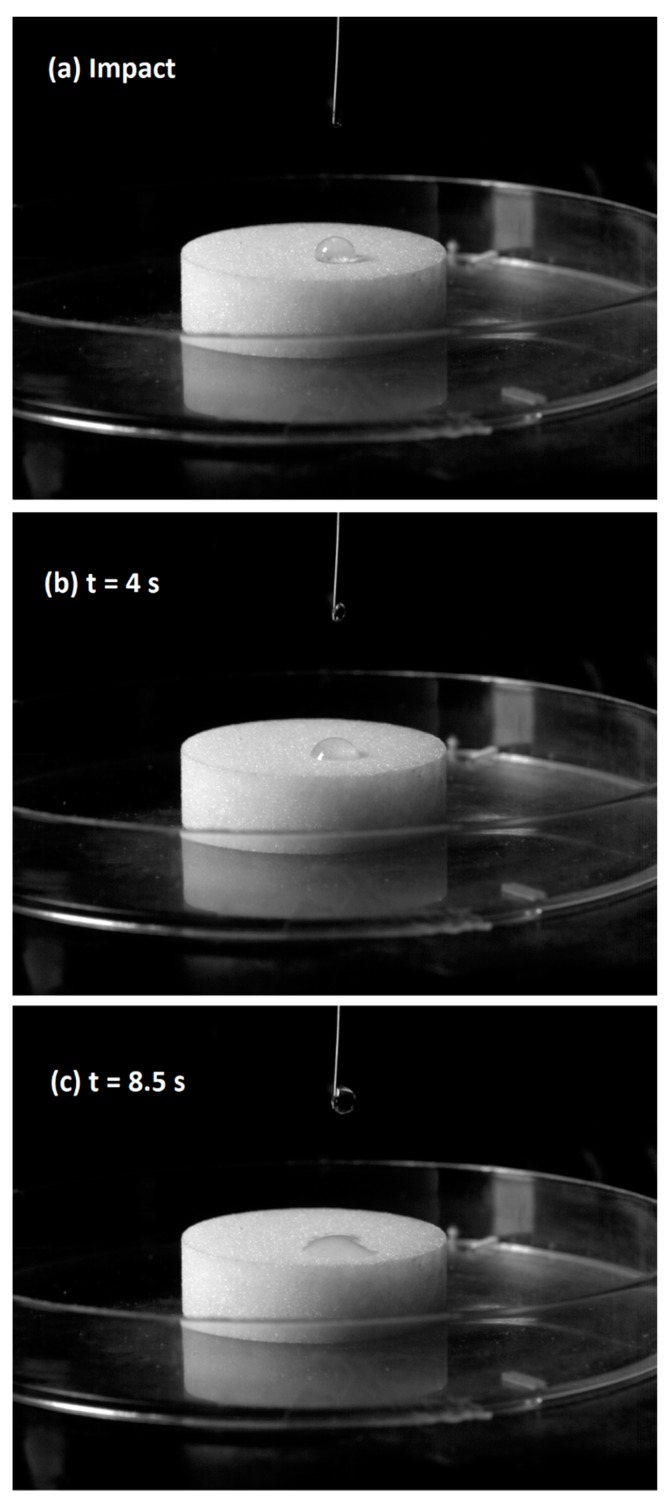
Orientation of USW droplet on urea surface at different penetration times: (**a**) impact, (**b**) t = 4 s, and (**c**) t = 8.5 s.

Penetration time is proportional to liquid viscosity and inversely proportional to adhesion tension. This emphasizes the importance of both wetting thermodynamics and kinetics. The contact angle needs to be less than 90° to ensure penetration. Figure 6 illustrates B3 droplet orientation at different penetration times. Both surface wetting and spreading are necessary since binder droplet cover very small surface area thereby restricting the number of collision that yield growth. Table 6 shows penetration time for different concentrations of USW binder.

Penetration time increases with increase in viscosity for higher concentration of the USW droplet. Consequently, probability of successful agglomeration also increases. When a single droplet hits the surface of powder, the droplet is suction driven into the powder pores by capillary forces [48]. Capillary forces highly affect the strength of wet agglomerate as well as viscous forces that contribute to the strength of granulates [47].

#### 3.1.5. Liquid Bridge Bonding Forces

**Figure 7:**
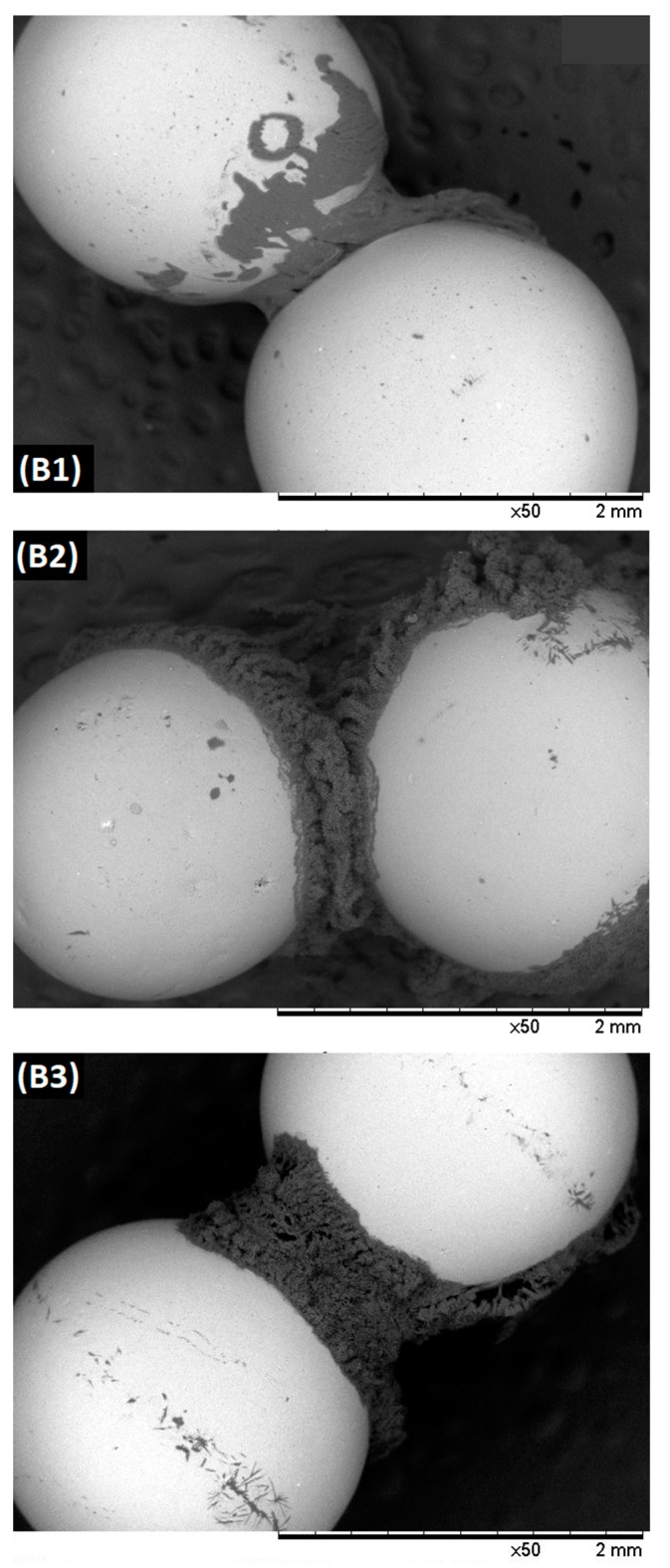
Liquid bridge bonding between two binary glass bead particles (2 mm) using different concentrations of USW binder (**B1**–**B3**).

Figure 7 represents liquid bridge between spheres of equal size (2 mm) after solidification of liquid for three different concentration of the USW binder (B1, B2 and B3). Binder volume between aggregated particles increases by increasing viscosity that is evident from the accumulation of the largest volume of binder particles between two spheres in case of B3 binder solution. It is suggested that cohesive strength of the dynamic liquid bridge may exceed that of the static liquid bridge by at least an order of magnitude due to the additional energy dissipation resulting from binder viscosity [13,24,30,31]. Both the capillary and viscous contributions were found to significantly affect the bonding mechanism of colliding particles [13]. Table 7 represents static force (*F*_stat_), dynamic force (*F*_dyn_), and capillary viscous number (*Ca*_vis_) for three different concentration of the USW binders. 

The difference between Equations (1) and (3) is in order of 0.05 that indicates that either of these equations can be used to determine the static force. *Ca*_vis_ number is divided into three levels; (1) If *Ca*_vis_ ≤ 10^−3^, the dynamic bridge strength is of the order of a static bridge and is insensitive to liquid viscosity. As a result, the strength of the dynamic pendular bridge is a superposition of Laplace-Young capillary and viscous dissipation forces [22], (2) If 1 ≤ *Ca*_vis_ ≤ 100, the bridge strength is sensitive to viscous forces, and (3) In granulation process the capillary contribution to the pendular bridge force can be neglected if 1 ≤ *Ca*_vis_ ≤ 100. In contrast, generally low-viscosity liquids are employed for coating operations and consequently the role of viscous forces becomes secondary [22]. 

Table 7 suggests that the value of *Ca*_vis_ increases by increasing the binder viscosity. For all three binder concentrations, 10^−2^ < *Ca*_vis_ < 1 and based on the aforementioned three categories, the *Ca*_vis_ achieved in this study is sensitive to both capillary and viscous forces.

**Figure 8:**
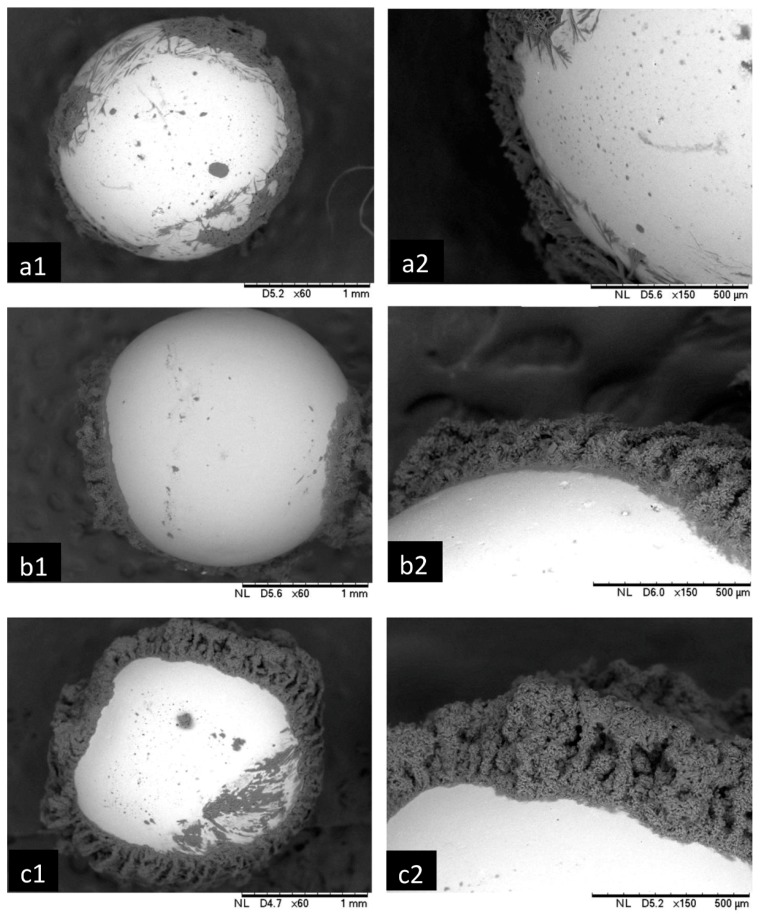
Schematic of spheres covered with binder. (**a1**) Covered with B1, (**b1**) covered with B2 and (**c1**) covered with B3. (**a2**), (**b2**) and (**c2**) are magnifying the corresponded particles.

Figure 8 portrays the micrographic pictures of spheres covered with liquid layer of binder. SEM analysis shows more asperities on the surface of agglomerated particles. Figure 8a,c represent spheres bound with the lowest and highest viscosities respectively. Viscosity has noticeable effect on atomization behavior of liquid and the resultant droplet size [22]. Due to strong cohesive force of B3 binder, thickness of layer is higher in comparison to B1 and B2 binders. Consequently, probability of successful collision increases for the particles covered with thicker layer due to significant role played by the viscous dissipation force [22]. By using Figure 8, thickness of layer and asperities are measured for each of the binders. To estimate probability of successful coalescence, viscous Stokes number (*St*_v_) and critical Stokes number (*St*_v_*) are calculated. An average velocity of particles (10 cm/s) was determined by high speed camera. 

Table 8 represent Stokes number for various particle sizes with respect to binder concentration. St number increases by increasing the particle size and decreases by increasing the viscosity. 

Based on our calculations, the critical St number, *St*_v_*, for B1, B2, and B3 resulted as 13.73, 13.81, and 15.2 respectively. Successful coalescence happens when *St*_v_ < *St*_v_*. The increase in binder viscosity increases the probability of successful coalescence. Binder with low viscosity (B1) tends to produce granulated particles with a maximum size of 2 mm but high viscosity binder can produce granulated particles as large as 4 mm. Viscous St number for B2 and B3 binders for all particle sizes are lower than critical St number and all collisions are considered as successful coalescences. Furthermore, even bigger (>5 mm) granulates are also formed by continuous spraying when B2 and B3 binders are used.

### 3.2. Effect of Operating Parameters on Particle Growth

#### 3.2.1. Statistical Analysis (ANOVA)

The result of analysis of variance (ANOVA) for the effect of fluidized bed operating parameters on the response objective (particle growth) is presented in Table 9. *p*-value is usually used to identify the statistically significant factors. A *p*-value less than 0.05 indicates model terms are significant [27]. Based on the ANOVA results, the Model *F*-value of 14.15 (*p*-value = 0.0001) implies that the model is significant. In addition, the *p*-values is less than 0.05 which indicate the model terms are significant. In this case A, B, C, AB, AC and AE appear as the statistically significant model terms while BD and DE are considered as marginally significant terms due to slightly higher *p*-value. The fluidizing air velocity (B) and primary urea weight (C) with a *p*-value of <0.0001 each are the most influential parameters. The "Curvature *F*-value" of 3.73 implies that the curvature (as measured by difference between the average of the center points and average of the factorial points) in the design space is not significant relative to the noise. The "Lack of Fit F-value" of 1.40 implies the Lack of Fit is not significant relative to the pure error. A *p*-value of <0.0001 indicates that the model is significant. The “Pred R-Squared” of 0.7148 is in reasonable agreement with the “Adj R-Squared“ of 0.8448. “Adeq Precision” measures the signal to noise ratio and a ratio greater than 4 is desirable. The AdEquation Precision of 14.158 indicates an adequate signal. 

Hence, this model can be used to navigate the design space. The residual versus predicted value and normal probability plots are two significant graphical approaches to check the validity of the regression model [43,49]. The results of the residual versus predicted value indicate the difference between predicted and observed values. 

**Figure 9:**
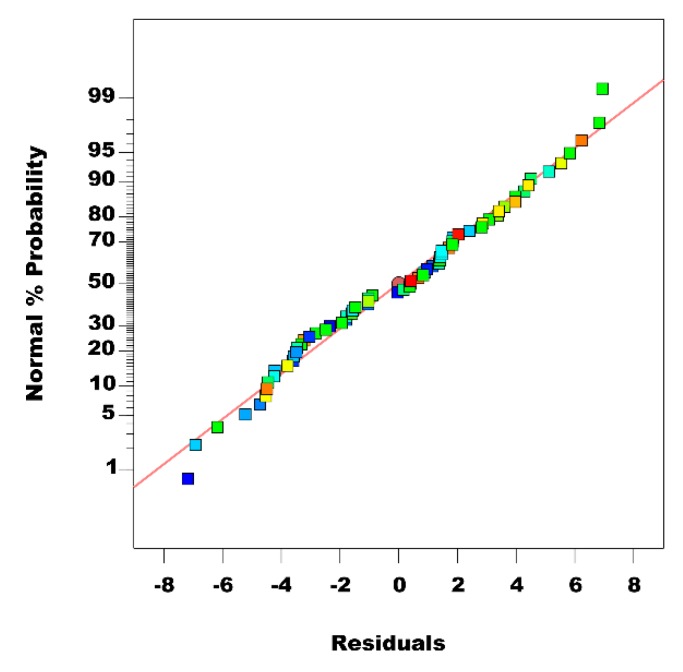
Normal plot of residual.

If the residuals have a regular pattern, it infers that the suggested model is not adequate. Moreover, residuals in normal probability plot should be laid in a straight line [50,51]. For the current study, the straight line in the normal plot of residuals in Figure 9 confirms that the model is adequate and correct.

#### 3.2.2. Interactive Effect of Air Temperature and Velocity on Particle Growth

**Figure 10:**
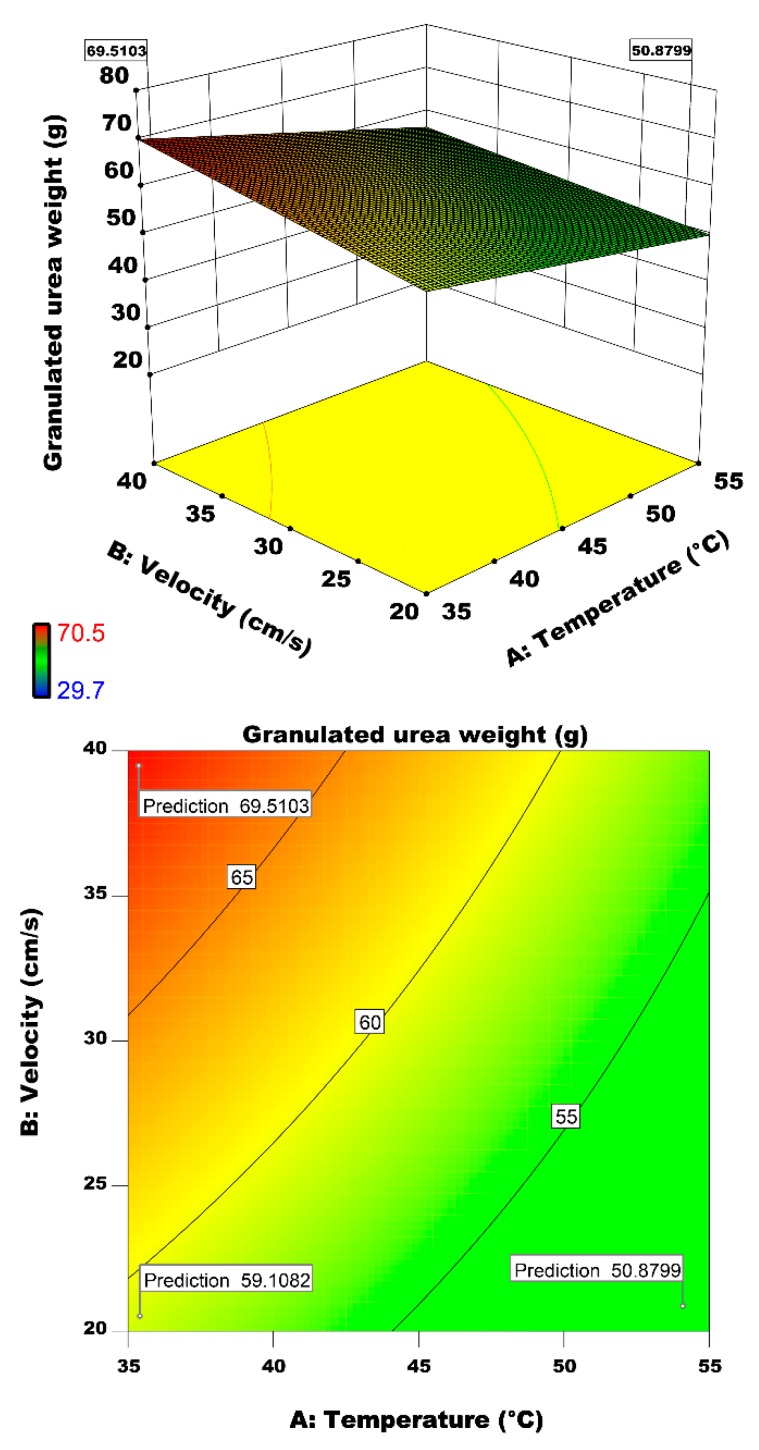
Effects of inlet air temperature and air inlet velocity on the granulated urea weight.

In order to find the optimum value of each factor, the two-dimensional (2D) and three-dimensional (3D) response surface contour plots are used. Growth of urea particles in terms of granulated urea weight as a response objective is illustrated in these figures. Only those combinations of the operating parameters on response objective are discussed that are statistically significant or marginally significant. Figure 10 illustrated the interactive effect of factors fluidizing air temperature and air velocity on the urea particle growth. Air inlet velocity and air inlet temperate are statistically significant factors as evident from their *p*-value (Table 9). The air velocity is a parameter that influences both the operating stability and granulation parameters [22]. The fluidization velocity is a crucial parameter in the control of the growth of the particles [29]. However, by changing one of these factors, the response will change enormously. 

Figure 10 shows maximum performances of the granulation process at highest velocity and lowest temperature at which we achieved the largest weight of the granulated urea particles. A comparison of our findings with the results of Dimin et al. [26] and Andrade et al. [6] vindicate that number of granulated particles increase by increasing air velocity. It is easier to produce particles with specific size by adjusting air velocity. By moving across the velocity coordinate from 20 to 40 cm/s, the lifting (buoyancy) and drag forces of air inlet is increase. In this case, the particles are easily pushed toward top of chamber even 1–2 mm particles. This led us to reach large particle size of about 4 mm. Minimum fluidization velocity for particles in the size range of 1–2 mm is about 20 cm/s. At air inlet velocity of about 40 cm/s, the probability of collision between granulated particles is increased and particles have a chance to stick to each other in the presence of a liquid bridge. The binder droplets from liquid bridge again and bigger particles are formed with this process of droplet impingement, liquid bridge formation, and sticking of new particles in these liquid bridges formed over a period of time till the growth of particles to the desired range. In this condition, higher predicted value for the granulated urea weight is 69.51 g. By reducing the air velocity, the granulated urea weight reaches to below 60 g. At a constant temperature of 35 °C, the particle growth reduces by 14% when the air velocity is reduced from 40 to 20 cm/s. In case of the inlet air temperature, the particle growth decreases by 17% when the temperature increases from 35 to 55 °C at a constant air velocity of 40 cm/s. Lowest growth of urea particles is observed at the air velocity of 20 cm/s and a temperature of 55 °C. 

#### 3.2.3. Interactive Effect of Air Temperature and Primary Urea Weight on Particle Growth

**Figure 11:**
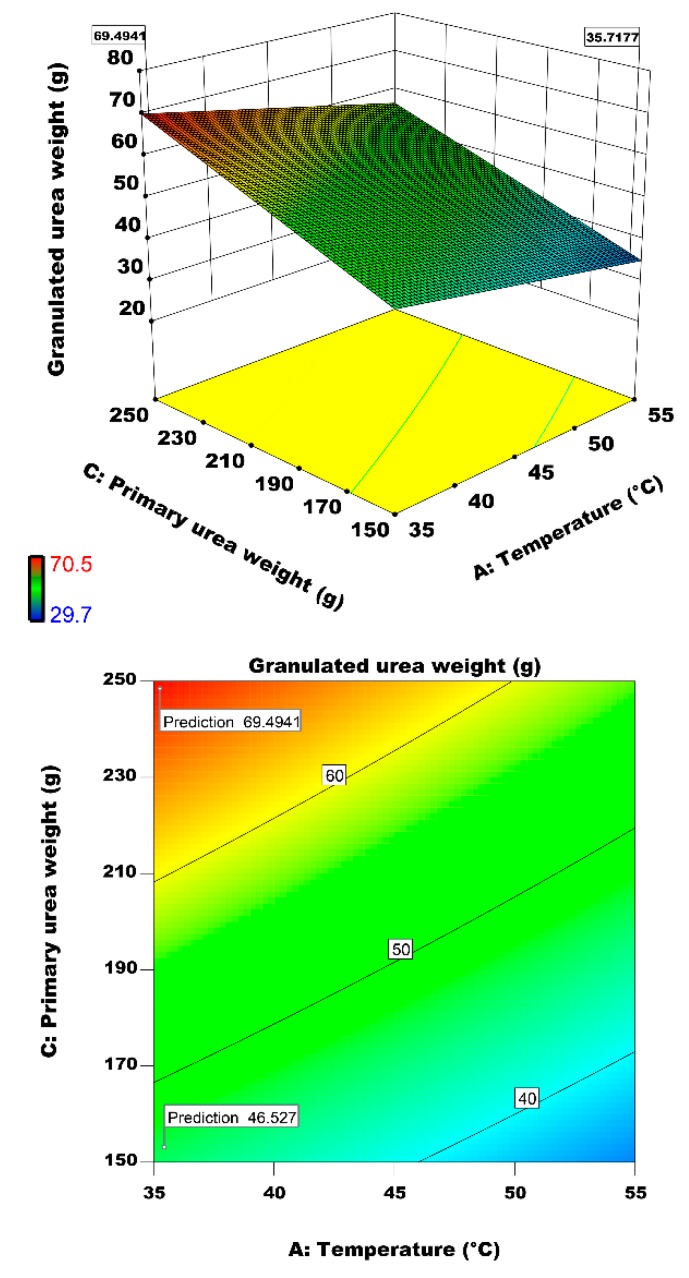
Effects of inlet air temperature and primary urea weight on the granulated urea weight.

Primary urea weight is another statistically significant factor that has a direct effect on granulation performance. Figure 11 represents the interactive effects of primary urea weight (substrate) and fluidizing air temperature on granulated urea weight. The results indicate that granulated urea weight increases by increase in primary urea weight and decrease in the air temperature. Studies of Chua et al. [7] on air inlet temperature is in-line with our finding. They reported that operating temperature should be adjusted to an optimum level. Very high or very low temperature has an adverse effect on granulation performance. The largest particle growth (69.49 g) is achieved at the air temperature of 35 °C and a primary urea weight of 250 g. the particle growth increases by 33% when we increase the primary urea weight from 150 to 250 g at a constant air temperature of 35°C. 

**Figure 12:**
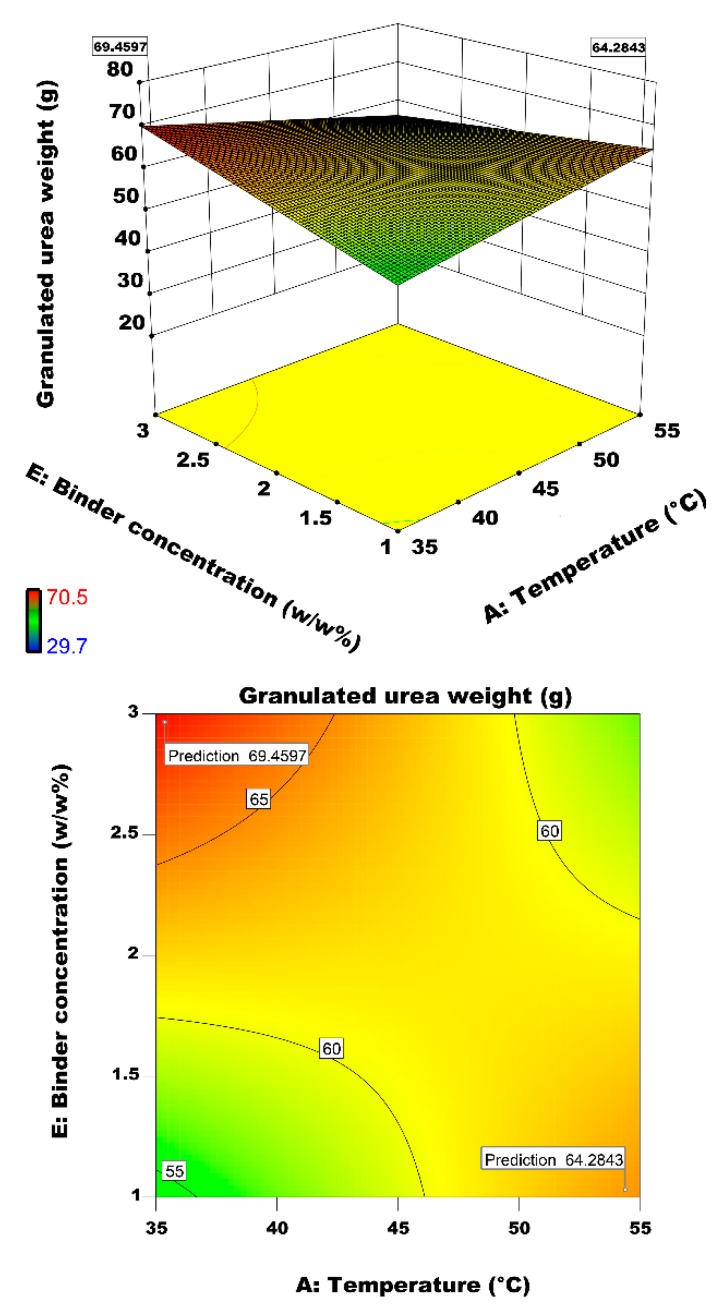
Effect of air inlet temperature and binder concentration on the granulated urea weight.

As illustrated in Figure 10 and Figure 11, air inlet temperature has an inverse effect on granulation performance. This is because of the premature drying of the binder droplet surface. Due to rapid evaporation of solvent from the droplet surface before collision, the probability of successful impingement and coalescence of droplet on solid urea particle reduces. Our results are in line with studies of Morl et al. [20] and Saleh and Guigon [22] on effect of wetting degree on agglomeration of particles. This undesired phenomenon of premature drying of the droplet surface at high temperature and subsequent relinquishment of the liquid bridge can be avoided by decreasing the binder concentration (see Figure 12) [52]. 

#### 3.2.4. Interactive Effect of Air Temperature and Binder Concentration on Particle Growth

Figure 12 illustrates the interactive effect of binder concentration and fluidizing air temperature on the granulated urea growth. Air temperature affects the binder concentration by evaporating solvent in it. After impingement of binder droplet on the particle surface, the binder concentration increases due to evaporation of the solvent. Subsequently, this increases the droplet viscosity that facilitates the liquid bridge formation. On the other side, we have observed in Figure 4 that binder viscosity decreases by increasing temperature, however, the binder concentration dominated viscosity due to fast evaporation of the solvent. It is observed in Figure 12 that the granulated urea weight is significantly high at: (1) highest concentration and lowest temperature, i.e., 69.46 g and (2) highest temperature and lowest concentration, i.e. 64.28 g. in the first case, the highly concentrated binder droplets are likely to experience the delayed evaporation after their successful impingement on the solid particle that facilitates the quick coalescence and subsequent larger growth of particles. In case 2, the augmented temperature makes the solvent evaporate at a relatively faster pace increasing thereby the concentration and, therefore, the viscosity of the binder droplet. Higher viscosity facilitates the growth of particles are discussed in the earlier section.

#### 3.2.5. Interactive Effect of Air Velocity and Primary Urea Weight on Particle Growth

**Figure 13:**
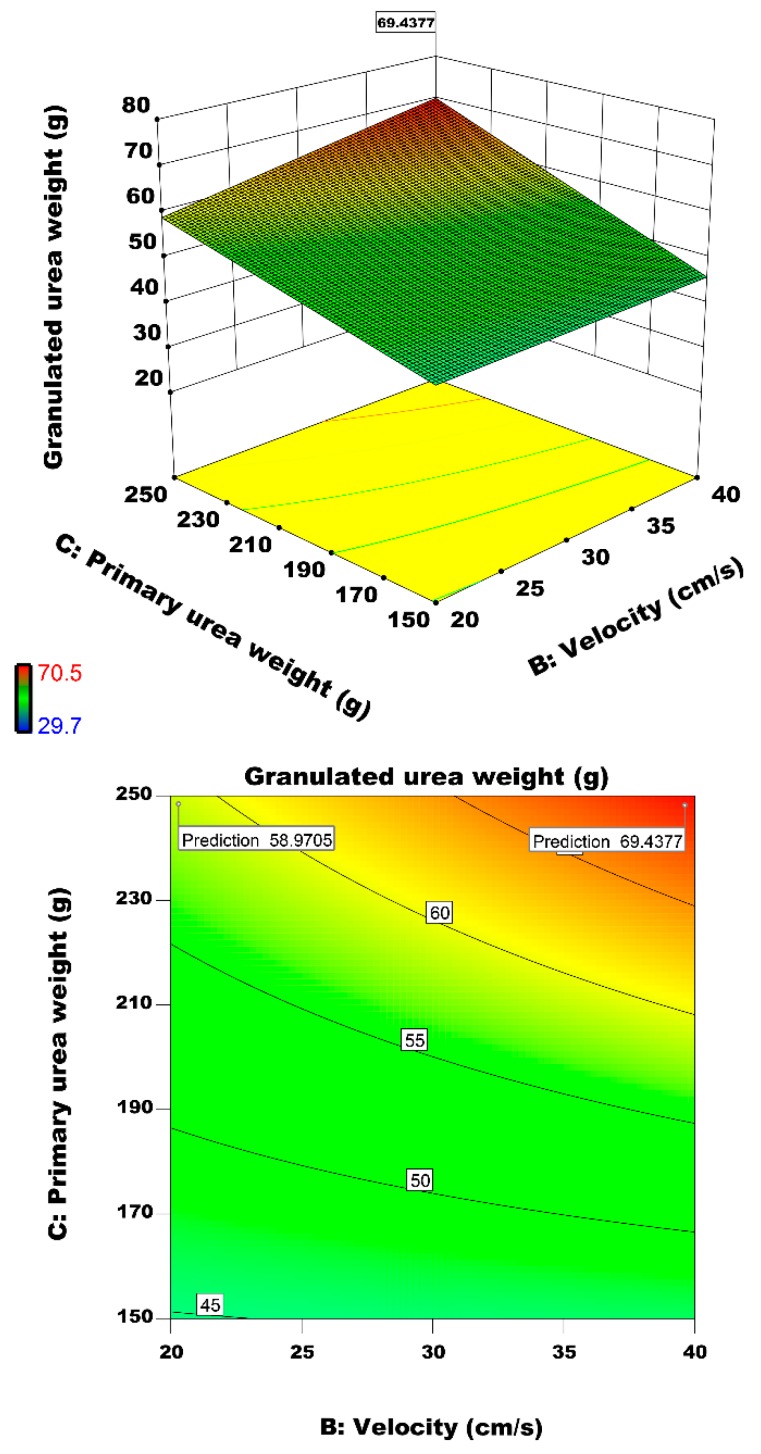
Effect of air inlet velocity and primary urea weight on the granulated urea weight.

Figure 13 represent primary urea weight and velocity on the granulated urea weight. Maximum weight of 69.43 g is predicted at primary urea weight and air inlet velocity of 250 g and 40 cm/s, respectively. The primary urea weight causes increase in residence time and collision. Saleh and Guigon [22] studies about weight of primary loaded particles demonstrate great consistency with our observations. Iveson [12] reported that primary particle size and binder content are two major factors that alter the granulation performance. His findings are in-line with our result that indicate that binder content is significant factor to determine the production size. The main reason for this condition related to volume of formed agglomerated particles. In constant velocity of 40 and 20 cm/s by changing primary urea weight from 150 to 250 g, response will increase 32% and 22%, respectively. Low velocity led us to obtain low response due to agglomerated particles of size of 1–2 mm cannot fluidized again and conclusively amount of granulated urea particles in range of 2–4 mm will be reduced. Considering the primary urea weight charge capacity of the fluidized bed, higher initial lead to higher amount of granules however higher initial charge of urea may lead to some operational problems, for example, clogging of fine powder filter elements installed on top of the fluidized bed. 

#### 3.2.6. Interactive Effect of Air Velocity and Binder Volumetric Flow rate on Particle Growth

**Figure 14:**
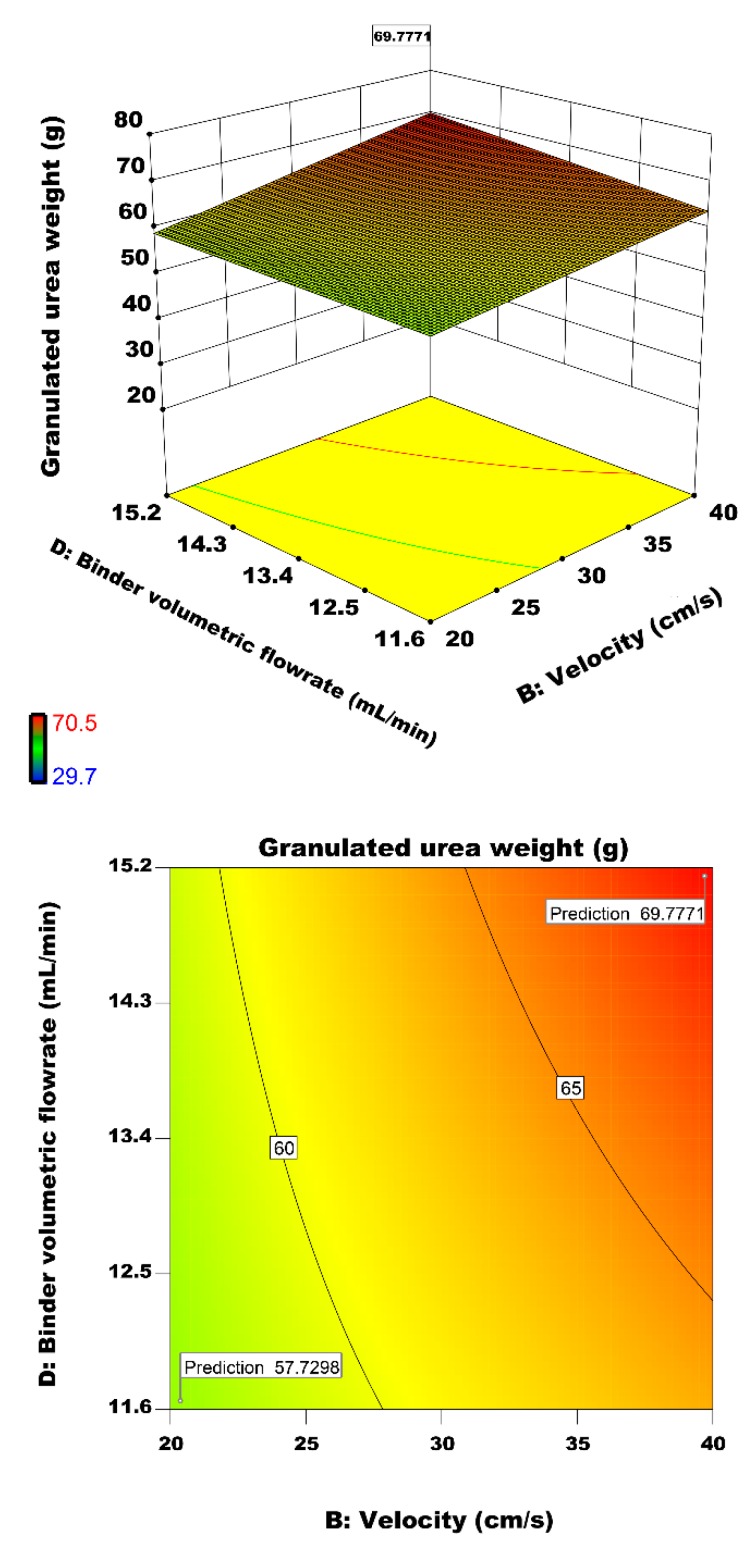
Effect of binder volumetric flow rate and air inlet velocity on the granulated urea weight.

In fluidized bed spray granulation, a permanent wetting of the particle surface and simultaneous evaporation and drying of the deposited liquid occurs [20]. An increase in binder spray rate increases particles’ wetting and subsequently enhances the granulation performance. Figure 14 illustrates the interactive effect of binder volumetric flow rate and fluidizing air velocity on the granulated urea weight. Both of these factors have a direct on the growth rate of granulated particles. The increase in binder spray rate and air velocity increases the granulated urea weight. At a lower air velocity, the spray rate doesn’t have a significant effect on particle growth. It is observed in Figure 14 that particle growth does not change much with increase in spray rate at a constant air velocity of 20 cm/s. However, the granulated particles’ weight hits the maximum value of 69.77 g by increasing both velocity and binder flow rate. At the air velocity of 40 cm/s, the increase in wetting of the particles led to more successful coalescence. 

**Figure 15:**
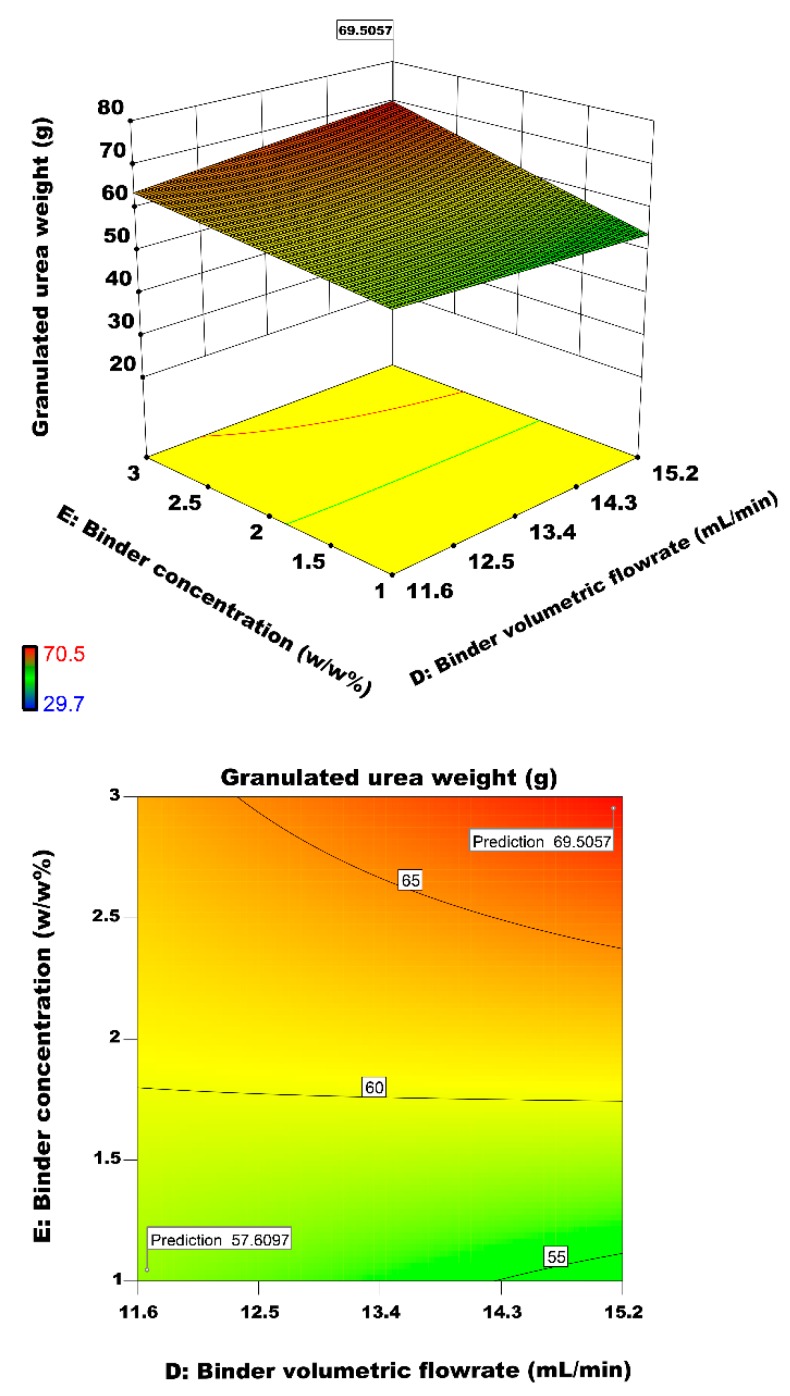
Effect of binder volumetric flow rate and binder concentration on the granulated urea weight.

The rate of top spraying is significant to control bed humidity and the granulated urea particle size [53]. Binder flow rate is statistically significant akin to findings of Andrade et al. [6]. Figure 15 represents effects of binder concentration and binder volumetric flow rate on granulated urea weight. The wetting dynamics control the agglomeration rate. At a higher wetting rate, the drying rate depends on air inlet temperature and heat transfer. For a lower spray rate, more time is required to reach to desire particle size. Binder with high viscosity has a strong tendency to agglomerate. The increase in binder flow rate increases the possibility of successful collisions and decreases the brittleness. Based on the main objective of this experimental design and the local optimum point of the model, the optimum value of the response objective (granulated urea particle) is 69.70 g. At the highest level of the surface inclination, the maximum granulated urea weight is achieved at the air inlet velocity of 40 cm/s, primary urea weight of 250 g, binder volumetric flow rate of 15.2 mL/min, binder concentration of 3 *w*/*w*%, and the air inlet temperature of 35 °C. 

## 4. Conclusions

In this study, the effect of starch based biodegradable binder properties and fluidized bed operating conditions on particle growth of granulated urea is investigated. A full factorial design of experiments is used to evaluate the statistically significant parameters and the interactive effect of these parameters on particle growth. Based on the results, binder with higher concentration demonstrates higher viscosity and higher penetration time, which potentially enhance the granulation performance. At the same time, the viscous Stokes number for binder with higher concentration is lower than critical Stokes number, which led to a higher successful coalescence rate. Furthermore, due to higher liquid viscosity and lower restitution coefficient of urea particles, the elastic losses in urea particle will takes place and contribute to successful coalescence. Air inlet velocity, air temperature and weight of loaded primary particles have major effects on granulation performance. Higher air velocity increases probability of collision, whereby lower temperature prevents binder to be dried up prior to the collision. In the future work, the optimum granulation operating parameters (evaluated from current work) would be used to model the granulation kinetics of urea using biodegradable binder.

## Figures and Tables

**Table 1:** Weight percentage of binder solution.

Solution No.	Starch Concentration (*w*/*w*%)	Urea Concentration (*w*/*w*%)
B1	1	49
B2	2	48
B3	3	47

**Table 2:** Factors and levels for response.

Factors	Symbols	Levels		
		Low Level (−1)	Center Point (0)	High Level (+1)
Air inlet temperature (°C)	A	35	45	55
Air inlet velocity (cm/s)	B	20	30	40
Primary urea weight (g)	C	150	200	250
Binder volumetric flow rate (mL/min)	D	11.6	13.4	15.2
Binder concentration (Urea *w*/*w*%)	E	49	48	47

**Table 3:** Binder density, viscosity, standard deviation, and standard error.

Solution No.	Binder Density (g/cm^3^)	Binder Viscosity (cP)	Standard Deviation (cP)	Standard Error (cP)
B1	1.14012	43	1.64	0.95
B2	1.13413	164	1.63	0.94
B3	1.12864	305	1.64	0.95

**Table 4:** Binder surface tension, standard deviation, and standard error.

Solution No.	Binder Surface Tension (N/m)	Standard Deviation (N/m)	Standard Error (N/m)
B1	58.41	0.422	0.24
B2	53.45	0.435	0.25
B3	47.13	0.431	0.24

**Table 5:** Contact angle, standard deviation, and standard error for USW binders.

Solution No.	Contact Angle (°)	Standard Deviation (°)	Standard Error (°)
B1	41.7	1.14	0.658
B2	73.85	0.629	0.363
B3	74.35	0.12	0.069

**Table 6:** Penetration time, standard deviation, and standard error for USW binder.

Solution No.	Penetration Time (s)	Standard Deviation (s)	Standard Error (s)
B1	1.95	0.15	0.086
B2	3.55	0.33	0.19
B3	8.5	0.70	0.40

**Table 7:** Static and dynamic force of liquid bridge bonding and capillary number for three concentrations of USW binder.

Solution No.	*F*_stat_	*F*_dyn_	*Ca*_vis_
B1	0.2729	0.00391	0.013
B2	0.378	0.006	0.016
B3	0.207	0.00762	0.036

**Table 8:** Viscous Stokes number for different particle sizes and binder concentrations.

Particle Diameter (µm)	Viscous St Number, *St*_v_		
	B1	B2	B3
250	2.062016	0.54065	0.29071
500	4.124031	1.081301	0.581421
1000	8.248062	2.162602	1.162842
2000	16.49612	4.325203	2.325683
3000	24.74419	6.487805	3.488525
4000	32.99225	8.650407	4.651366

**Table 9:** The results of ANOVA for particle size.

Source	Sum of Square	Degree of Freedom	Mean Square	*F*-Value	*p*-Value
Model	7457.94	27	276.22	14.51	<0.0001
A	167.77	1	167.77	8.81	0.0050
B	613.43	1	613.43	32.22	<0.0001
C	3852.06	1	3852.06	202.31	<0.0001
D	34.93	1	34.93	1.83	0.1832
E	1.60	1	1.60	0.084	0.7734
AB	80.33	1	80.33	4.22	0.0466
AC	84.73	1	84.73	4.45	0.0412
AD	13.76	1	13.76	0.72	0.4003
AE	395.81	1	395.81	20.79	<0.0001
BC	40.26	1	40.26	2.11	0.1537
BD	68.48	1	68.48	3.60	0.0651
BE	30.36	1	30.36	1.59	0.2140
CD	0.15	1	0.15	7.886 × 10^−3^	0.9297
CE	29.35	1	29.35	1.54	0.2216
DE	61.11	1	61.11	3.21	0.0808
Curvature	71.00	1	71.00	3.73	0.0606
Residual	761.61	40	19.04	-	-
Lack of Fit	102.74	4	25.69	1.40	0.2525
Pure Error	658.86	36	18.30	-	-
R^2^ =0.9073, Adjusted R^2^ = 0.8448, Predicted R^2^ = 0.7148, AdEquation Precision = 14.158					
